# Fine-Scale Habitat Heterogeneity Influences Occupancy in Terrestrial Mammals in a Temperate Region of Australia

**DOI:** 10.1371/journal.pone.0138681

**Published:** 2015-09-22

**Authors:** Ingrid Stirnemann, Alessio Mortelliti, Philip Gibbons, David B. Lindenmayer

**Affiliations:** Fenner School of Environment and Society, The Australian National University, Canberra, ACT 260, Australia; Università degli Studi di Napoli Federico II, ITALY

## Abstract

Vegetation heterogeneity is an inherent feature of most ecosystems, characterises the structure of habitat, and is considered an important driver of species distribution patterns. However, quantifying fine-scale heterogeneity of vegetation cover can be time consuming, and therefore it is seldom measured. Here, we determine if heterogeneity is worthwhile measuring, in addition to the amount of cover, when examining species distribution patterns. Further, we investigated the effect of the surrounding landscape heterogeneity on species occupancy. We tested the effect of cover and heterogeneity of trees and shrubs, and the context of the surrounding landscape (number of habitats and distance to an ecotone) on site occupancy of three mammal species (the black wallaby [*Wallabia bicolor*], the long-nosed bandicoot [*Perameles nasuta*], and the bush rat [*Rattus fuscipes*]) within a naturally heterogeneous landscape in a temperate region of Australia. We found that fine-scale heterogeneity of vegetation attributes is an important driver of mammal occurrence of two of these species. Further, we found that, although all three species responded positively to vegetation heterogeneity, different mammals vary in their response to different types of vegetation heterogeneity measurement. For example, the black wallaby responded to the proximity of an ecotone, and the bush rat and the long-nosed bandicoot responded to fine-scale heterogeneity of small tree cover, whereas none of the mammals responded to broad scale heterogeneity (i.e., the number of habitat types). Our results highlight the influence of methodological decisions, such as how heterogeneity vegetation is measured, in quantifying species responses to habitat structures. The findings confirm the importance of choosing meaningful heterogeneity measures when modelling the factors influencing occupancy of the species of interest.

## Introduction

Heterogeneity (defined here as dissimilarity or variation in a given attribute of vegetation across space) characterises the habitat structure of most ecosystems, and influences the distribution of biota [1–3]. For example, according to habitat heterogeneity theory [4], biota are more likely to occupy highly heterogeneous habitats as these habitats provide greater fitness benefits and resources [5, 6]. The study of heterogeneity in ecology and biogeography is a diverse topic that has received considerable attention and motivated the quantification of many different measures of vegetation heterogeneity (e.g. number of habitats, variation in habitat features [7]). An understanding of how vegetation heterogeneity influences biota is needed to identify the factors that drive species-habitat relationships [8]. While knowledge of association between some measures of vegetation heterogeneity (e.g. number of habitats and foliage height diversity) and biota have be recognised for decades [4, 9], there is still only a limited understanding of the effect of different types of heterogeneity on the spatial distribution of different fauna.

At the fine-scale (10’s of meters), vegetation is typically measured in terms of its amount or cover [10, 11]. Fine-scale variation in vegetation cover (hereafter habitat heterogeneity) is rarely explicitly measured, despite being regarded as critical for explaining the distribution of biota [12, 13]. An understanding of habitat heterogeneity is important since variation in the configuration of vegetation at the fine-scale is thought to influence species patterns through their response to risks, such as predation [14], and access to resources [5, 6]. Therefore, quantifying vegetation heterogeneity, as well as the amount of vegetation cover is believed to be essential for understanding animal distribution patterns and for informing management decisions [15–17]. However, studies rarely quantify fine-scale habitat heterogeneity, possibly because robust measures of habitat heterogeneity are time consuming to gather in the field (i.e. requires multiple samples per plot). Therefore, the influence of habitat heterogeneity, as well as the amount of vegetation cover, on species occupancy is not well understood. Knowing if and how habitat heterogeneity and cover influences species occupancy is important for informing management decisions on how to manage understory and over-storey vegetation to make it more suitable for species.

The context and surrounding heterogeneity of a landscape also plays an important role in driving species distribution patterns [18–20]. For instance, the proximity of a site to an ecotone (a highly heterogeneous and diverse area where different vegetation communities coincide; [21]) has been found to influence small mammal occupancy (e.g. [22]). Moreover, the context of the embedded habitats (e.g. the surrounding number of habitat types, hereafter landscape heterogeneity) also can influence species richness [23]. The response of biota to ecotones has been studied under various levels of anthropogenic influence (natural, semi-natural; e.g. [22, 24]). In contrast, the majority of studies that have investigated the influence of landscape heterogeneity on biota have been restricted to landscapes under intense anthropogenic influence [25, 26].

Small- to medium-sized terrestrial mammals are an ideal group for investigating the influence of habitat heterogeneity and landscape heterogeneity. This is because mammals of this size have relatively low mobility (comparatively to other taxonomic groups [i.e. birds]) and thus have more restricted home range and habitat requirements [27]. Most small- to medium-sized terrestrial mammals also perceive the environment at small spatial scales [28, 29], which should make them particularly sensitive to both fine-scale vegetation structure and the surrounding landscape heterogeneity.

We conducted a multi-scale study to answer two questions: 1) What is the influence of the amount and heterogeneity of habitat on mammal occupancy? And 2) What is the influence of different forms of vegetation heterogeneity (fine-scale vegetation heterogeneity, landscape heterogeneity and distance to ecotones) on mammal occupancy?

To answer these questions, we investigated the factors influencing occupancy patterns of three small- to medium-sized mammals, which have different environmental needs. Our study environment within Booderee National Park in south-eastern Australia was ideal for this investigation because this area is a naturally heterogeneous, patchy and characterised by many distinct vegetation types. Geological and fire disturbance processes in this landscape are also spatially variable, resulting in varied amounts of fine- to broad-scale habitat heterogeneity within the study area [30].

Here we test the hypothesis by Louys et al. [12] and McElhinny et al. [13], that some habitat attributes influence the occupancy of biota in terms of their variance, or heterogeneity, in addition to the more common measure of amount of cover. Furthermore, based on the habitat heterogeneity theory [4], we hypothesise that species occupancy will exhibit a positive response to both habitat heterogeneity, landscape heterogeneity and proximity to ecotones. Theoretically, vegetation heterogeneity is an important driver of biodiversity because it captures the range of resources available in a given area [31].

## Materials and Methods

### Ethics statement

Our study was observational and no plants or animals were harmed. The project was conducted in accordance with the requirements of permit CRE60.09 issued by the Animal Experimentation Ethics Committee of The Australian National University. We also obtained a Permit for Research in a Commonwealth Reserve (BDR10/00010).

### Study area

**Fig 1:**
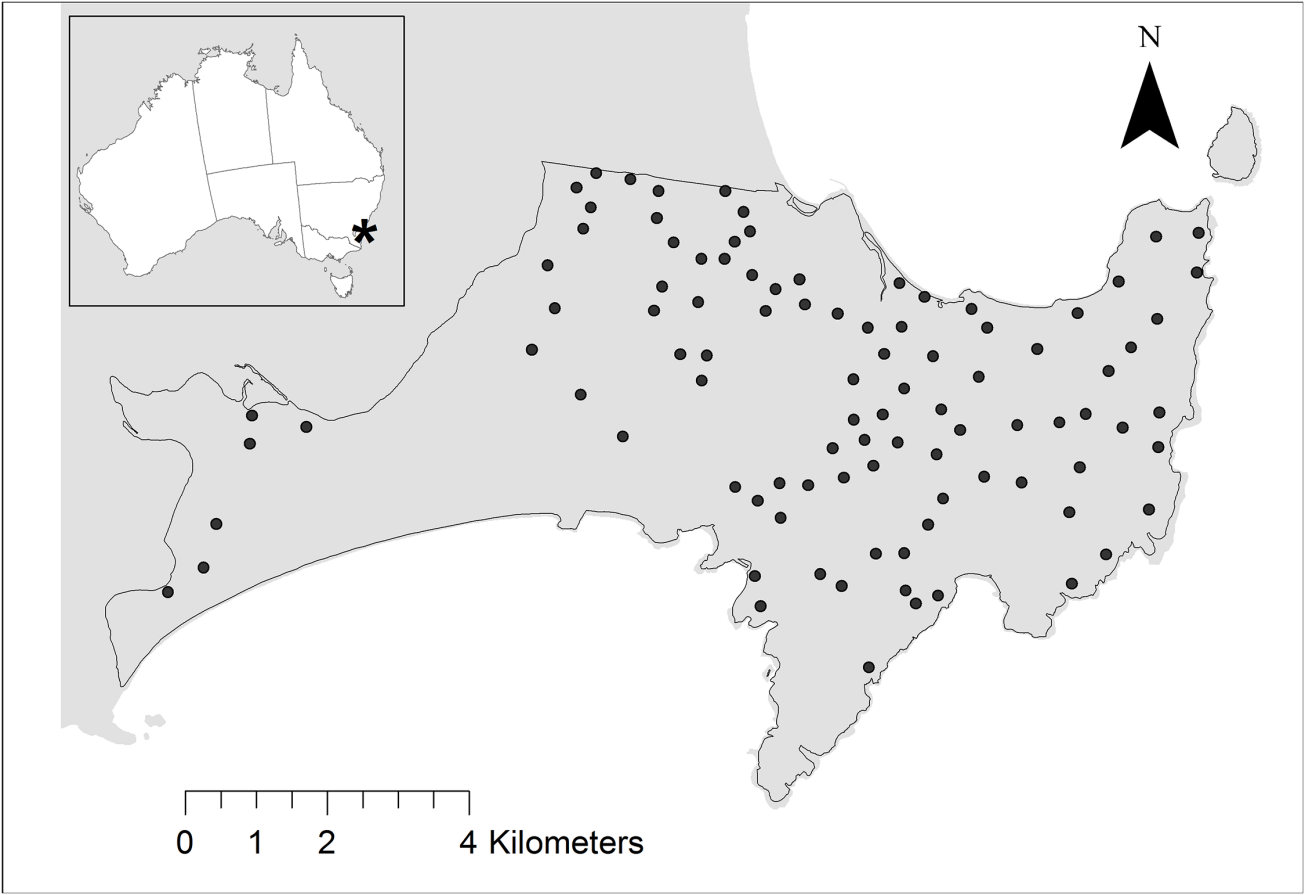
Our study was located within Booderee National Park on the south coast of New South Wales, south-eastern Australia. Black points are the study sites (*n* = 96).

Our study was located within Booderee National Park (35°10′ S latitude, 150°40′ E longitude) in the Jervis Bay Territory in south-eastern Australia (Fig 1). Booderee National Park is a lowland region (< 170 m ASL) of sandstone bedrock overlain by varying depths of deposited sand [32]. This area is characterised by a temperate maritime climate, with average rainfall of approximately 1200 mm that is largely consistent over the year. Mean annual temperature ranges from 17°C to 26°C. Booderee National Park is characterised by an extreme and unique degree of broad- and fine-scale vegetation heterogeneity within a fairly small area (~ 6500 ha). Therefore, potential confounding influences on mammal presence associated with climate and topography are minimised. Booderee National Park contains a variety of vegetation types, from dry heathlands to wet rainforest, which are patchily distributed within the park [33, 34]. At the fine-scale, the varied terrain, topography and fire history of the park has resulted in a high degree of variation in the different types of structural vegetation (e.g. trees and shrubs) within vegetation patches. For our study, we recognised four broad categories of vegetation (forest, woodland, shrubland, and heathland) and 29 vegetation sub-formation classes (S1 Table). The 29 vegetation sub-formation classes cover a wide range of natural vegetation, including: littoral rainforest, *Eucalyptus paniculata* dry sclerophyll forest, *Backhousia myrtifolia* dry rainforest, *Eucalyptus gummifera* dry woodland, *Avicennia marina* mangrove woodland, *Baeckea imbricata* coastal heath, and *Allocasuarina* dry shrubland. There are 12 forest groups, 7 woodland groups, 5 heathland groups and 5 shrubland groups. Full profiles of each of the 29 vegetation sub-formation classes are provided in Taws [34].

Booderee National Park has a complex history of both natural and prescribed fires. Spatially-explicit records of the fire history of the Jervis Bay Territory date back to 1937 [35].

### Study design

We used a stratified design to investigate the occurrence of small mammals in relation to fine-scale differences in the amounts of vegetation heterogeneity and vegetation cover. We stratified the study area into three of the vegetation types (forest, woodland and heathland), two fire frequency categories (0–3 years; 4–8 years), and two slope categories (low [0.24–3.56 degrees] and high [3.57–15.02 degrees]) (S2 Table). These three factors are known to influence both the degree of vegetation heterogeneity and amount of vegetation cover within the landscape (e.g. [36–39]). We selected eight (25m radius) sites within each of the 12 stratification treatments giving 96 sites within the study area. Our stratified approach enabled us to maximise the range (i.e. very low to very high values) of both heterogeneity and cover of different vegetation variables across our sites.

### Study species

We focused on three target species in our study the: black wallaby (*Wallabia bicolor*), long-nosed bandicoot (*Perameles nasuta*), and bush rat (*Rattus fuscipes*). All species are currently classified as least concern by IUCN red data list [40] and are distributed widely along the east coast of Australia [41]. The black wallaby is a macropod marsupial that weighs about 10.3–20.5 kg [41]. It is predominantly a browser and consumes a wide variety of plant and fungus species [42]. The long-nosed bandicoot is an omnivorous marsupial that weight ranges from 85–1100 g [41]. Its diet is primarily invertebrates, succulent plant material, and fungi [43]. The bush rat is an omnivorous mammal that weighs about 200g [44] and eats plant material, fungi and invertebrates [45].

### Habitat data

We selected 10 fine- and broad-scale explanatory variables that that are considered to influence the distribution of small- and medium-sized mammals [13, 46, 47]. We divided these explanatory variables into two categories, those characterising structural habitat at a fine-scale (within a 25m radius), and those characterising broader context of the surrounding landscape (within a 50m, 100m and 150m radius).

### Fine-scale habitat variables

Our fine-scale habitat variables were measures of the amount and variation of important structural habitat attributes at this scale [13]. To obtain these variables at each of our 96 sites, we collected data on three habitat attributes: shrubs (0–4 m); small trees (4–10 m) and tall trees (>10m). Each site consisted of a circular plot with a 25 metre radius. Within each site, we established nine subplots. Subplots were evenly distributed into three distance categories from the centre: 1) 4 to 9 m, 2) 12 to 17 m, and 3) 20 to 25 m. Subplots also were evenly located at 90, 210, or 330 cardinal degrees from the central point. Each subplot consisted of six sampling points, each spaced one metre apart. At each of the 54 sampling points per site, we measured the presence/absence of the three habitat attributes using the point intercept method [48].

We calculated two fine-scale measurements for each of the three habitat attributes: 1) a measure of the total percent cover and, 2) a measure of heterogeneity. To quantify each measure of total percent cover (hereafter called ‘cover’), we calculated the proportion of presences out of the total (*n*_*points*_ = 54) of each of the three habitat attributes.

Habitat heterogeneity is a measure of the variation of vegetation cover within a patch. In this study, we defined habitat heterogeneity as a measure of the differences in the: (1) proportion cover and/or (2) spatial dependency of habitat attributes, among the nine sub-plots within each site. Higher spatial dependence and/or increased difference between sub-samples within a site denote higher vegetation heterogeneity.

Our measure of fine-scale vegetation heterogeneity was derived by fitting a logistic regression model (a generalised linear model with a binomial distribution) to the cover data for each site. The response variable was the number of times (out of six) each feature was present, for each of the nine sub-plots (see above) for each site. We did not include any predictor variables in the model as the goal was to assess the adequacy of the model constant in describing the percent cover across the nine-subplots. We assessed the adequacy of the model by dividing the residual deviance by the degrees of freedom (in this case, d.f. = 8) which can be interpreted as a measure of over-dispersion. We used this measure of over-dispersion as our measure of heterogeneity at the site level. This approach was preferred to the usual measures of heterogeneity (such as the coefficient of variation) as it respects the underlying binomial structure of the data [49].

### Broad-scale habitat variables

To calculate broad-scale heterogeneity measures, we created a radius around each of the central points of each site at 50 m, 100 m, and 150 m (hereafter referred to as buffers). Within each buffer, we calculated the number of vegetation types from a detailed vegetation layer of the study site (see [34]) using Arc GIS [50]. This measure of landscape heterogeneity represents the degree of landscape fragmentation surrounding each site (i.e. as the number of habitats increase the habitat fragmentation in the area will also increase) [8]. To retain the nested structure between each buffer, but reduce collinearity between buffers, we recast each variable as a linear combination of the variable (i.e. the 150m extent remains the same, while the 100 and 50m extents are recalculated as the difference between the original variable and the one it is nested within; [51]).

To calculate the distance to the ecotone, we measured the Euclidean distance from each site to the closest ecotone (transition zone between vegetation communities) using Arc GIS. The nearest ecotone was deemed to be the closest habitat sub-formation based on detailed vegetation maps of the study area (see [34]). Arc GIS operations were calculated in Arc-View GIS version 9.2 [50].

### Camera trapping protocol

We used remote trail camera models with passive infrared sensors (either Scoutguard SG550® or Reconyx PC90®) to detect small- to medium-mammals at each of our 96 sites. Each camera was mounted approximately 50 cm above the ground at the centre of each site. A bait station was placed approximately 2.5 m in front of each camera. A small amount of peanut butter mixed with oats was secured to the ground under a vent cowl, as a lure at each site [52]. We sampled twenty-four sites continuously for eight consecutive nights. Twelve sites were sampled with Reconyx cameras and 12 sites were sampled with Scoutguard cameras. The twenty-four cameras (12 Scoutguard and 12 Reconyx) were rotated between sites until all sites had been surveyed with both cameras. All camera monitoring occurring within April-May 2011.

### Statistical analysis

We used occupancy models to determine the factors influencing species occupancy as these models can correct for imperfect detection due to false absences (i.e. failure to detect a species that is present at the site; [53]). False absences are common in fauna studies, with detection probability typically being less than one in field conditions (e.g. [54]).

In our models, a site (sensu [55]) was defined as a camera-trap station. In our study a “visit” to a site was any record of each species within a 24-hour time period (i.e. from midday to midday). The response variable in our analysis was the detection history of each mammal species per site, which is the sequence of visits (1’s and 0’s) over the complete survey period (eight days per site).

#### Detection covariates

We identified four detection covariates (three continuous and one categorical variable) that we hypothesised could cause variation in species detection probability (*p*): type of remote camera (camera type), vegetation type (e.g. tall tree cover), heterogeneity of shrub cover and the total shrub cover (S3 Table). Previous studies have suggested that different camera models differ in their ability to detect mammals [56, 57]. The amount of understorey (e.g. cover of shrubs) also affects the intensity of use of an area by small mammals [58, 59] and thus may influence detection probability. Spatial heterogeneity in the environment (i.e. shrub heterogeneity) also may influence detection probability, as it can influence the movement patterns of small mammals [60]. Detection probability may also be lower within particular vegetation types. In our study, we used tall tree cover as a continous proxy of vegetation type. To account for a detection bias, we incorporated these four variables (camera type, shrub cover, shrub heterogeneity, and vegetation type) into the detection component of our models for each species.

#### Occupancy covariates

We measured ten site variables that we hypothesized could affect occupancy of our three target mammal species: three cover variables (shrub, small trees and larger trees), three heterogeneity variables (shrub, small trees and larger trees), the distance to an ecotone, and the number of habitats surrounding our sites (within 50m, 100m and 150m buffers; S3 Table). These ten covariates were chosen to represent key habitat types or limiting factors for these species.

Prior to analysis, we assessed collinearity in the all explanatory variables using pairwise scatterplots, and Variance Inflation Factors [61, 62] with the R software [63]. Variance Inflation Factors were all below three, suggesting our explanatory variables were not strongly collinear [51] and we therefore considered all variables in our occupancy models.

We used single-season models to model the probability of occupancy (ψ) of each mammal species per site, while accounting for detection probability (*p*) [55], within the software package unmarked [64]. We used single-season models as we assume the populations of all three of our study species were closed to any changes with respect to occupancy of the sampling sites during our sampling period [55]. Due to convergence issues within our full models, we also were unable to use a backward stepwise approach [65]. Thus, we used a multi-step process with a forward stepwise selection approach to determine the best ranked model [66]. First, we used forward stepwise selection to consider the effect of all single variables on species detection probability. We then tested all combinations of all single predictor variables that were ranked above the null model. We retained the top ranked parameterisation for detection probability and repeated the process with the occupancy predictor variables to determine the best occupancy model for each species. The ranking of each of the models were compared using Akaike’s Information Criteria adjusted for sample size (AICc) [67]. To account for uncertainty in model selection, we used a model averaging approach (i.e. we averaged all models within two delta AICc of our top model) to predict site occupancy [67].

## Results

We detected the bush rat at 13 of the 96 sites (14%); the long-nosed bandicoot was detected at 17 of the 96 sites (18%); and the black wallaby at 35 of the 96 sites (36%).

### Black wallaby

**Fig 2:**
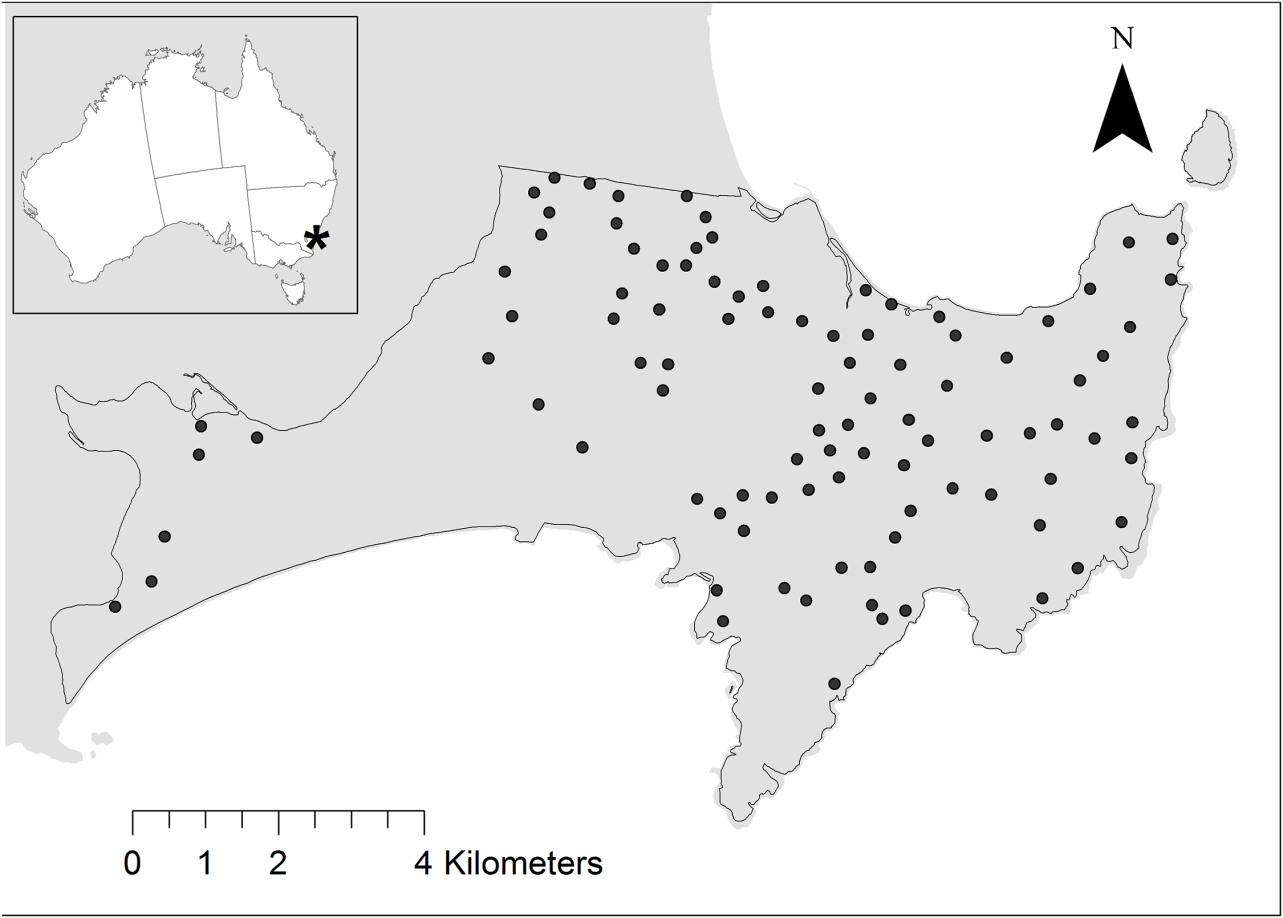
Probability of occupancy (± S.E.) of three mammals: black wallaby, bush rat, and long-nosed bandicoot in response to cover of small trees and heterogeneity of small trees, and distance to an ecotone.

The top ranked model predicting the probability of black wallaby occupancy contained distance from an ecotone in the occupancy component of the model and camera type in the detection component (Table 1). The probability of occupancy of the black wallaby had a strong negative association with distance from an ecotone (Fig 2). We found no evidence to suggest that either heterogeneity or cover of any of the fine-scale habitat attributes (shrubs, small trees or tall trees) influenced the probability of occupancy of the black wallaby. The probability of detecting black wallaby was affected by camera type, and was higher for Reconyx cameras, than for Scoutguard cameras (Fig 2). Two models predicting the probability of black wallaby occupancy had comparable support (i.e. were within two delta AIC of our top model; Table 2). The second ranked model predicting black wallaby occupancy contained all candidate terms contained in the top ranked model, except tall tree cover.

**Table 1:** Estimated parameters (β) and standard error (S.E.) of the top ranked occupancy models for black wallaby, long-nosed bandicoot, and bush rat.

Species	Parameter	Variable	β	S.E
Black wallaby	Ψ	Intercept	1.03	0.58
	Ψ	Distance to an ecotone	-16.13	7.62
	*P*	Intercept	-1.84	0.30
	*P*	Camera type	1.20	0.39
Long-nosed bandicoot	Ψ	Intercept	-1.04	0.72
	Ψ	Small tree heterogeneity	0.75	0.36
	Ψ	Small tree cover	-8.09	4.50
	*P*	Intercept	-1.86	0.40
Bush rat	Ψ	Intercept	-2.13	0.68
	Ψ	Small tree heterogeneity	0.44	0.19
	*P*	Intercept	-3.14	0.87
	*P*	Shrub cover	2.45	1.26

**Table 2:** Summary of the top ranked occupancy models (Δ AICc <2) and relative weights (*W*) for: black wallaby, long-nosed bandicoot, and bush rat. The terms in the parentheses represent the detection (*p*) and occupancy (ψ) covariates found in the models: camera type (cam), distance to an ecotone (E), small tree heterogeneity (STH), shrub cover (SC), small tree cover (STC) and tall tree cover (TTC). Absence of the ψ parameter in the model notation implies a constant model.

Species	Model	Δ AICc	*W*
Black wallaby	ψ(E,), *p*(CAM)	0	0.47
	ψ(E, TTC), *p*(CAM)	1.74	0.19
Long-nosed bandicoot	Ψ(STH, STC), *p*()	0	0.40
Bush rat	ψ(STH), *p*(SC)	0	0.52
	ψ(STH, TTC), *p* (SC)	1.60	0.23
	ψ(STH, STC), *p*(SC)	1.83	0.21

### Long-nosed bandicoot

The top ranked model predicting the probability of long-nosed bandicoot occupancy contained small tree heterogeneity and small tree cover in the occupancy component of the model, with the detection component held constant (Table 1). The probability of occupancy of the long-nosed bandicoot was highest in areas with a high degree of small tree heterogeneity and low amounts of small tree cover (Table 1 and Fig 2). None of the other models we tested had comparable support (i.e. were within 2 delta AIC of the best ranked long-nosed bandicoot model; Table 2).

### Bush rat

The top ranked model predicting the probability of bush rat occupancy contained small tree heterogeneity within the occupancy component of the model, and shrub cover within the detection component (Table 1). The probability of occupancy of the bush rat was highest in areas with high levels of small tree heterogeneity (Fig 2). The detection probability of the bush rat was highest in areas with high levels of shrub cover (Fig 2).

Two models predicting the probability of bush rat occupancy had comparable support (i.e. were within two delta AIC of our top model; Table 2). The second ranked model contained the same variables as the top ranking model, but also included an effect of tall tree cover. The third ranked model also contained same variables as the top model, but also included an effect of small tree cover.

## Discussion

We found empirical evidence to support our hypothesis that fine-scale heterogeneity of vegetation cover can influence mammal occupancy. To the best of our knowledge, no other study has explicitly tested for and established this relationship. Furthermore, we found different species varied in their response to different types of vegetation heterogeneity. For example, the black wallaby responded to proximity of ecotones, and the long-nosed bandicoot and bush rat responded to fine-scale habitat heterogeneity, whereas landscape heterogeneity appeared to have no effect on the probability of occupancy of our three study species. Our results highlighted the impact of methodological decisions such as how heterogeneity in vegetation is measured, in influencing species responses and the importance of choosing meaningful, heterogeneity measures for the species and study system of interest.

### What are the effects of fine-scale habitat heterogeneity and cover on occupancy?

We found, as hypothesised (but not explicitly tested) by Louys et al. [12] and McElhinny et al. [13], that some habitat attributes may influence the occupancy of biota in terms of their variance or heterogeneity rather than the more commonly used measure, absolute cover. For example, we found that small tree heterogeneity was included in the final model for the long-nosed bandicoot, and both heterogeneity and cover of small trees were included in the final model for the bush rat (Table 2). Understanding how variance (heterogeneity) of important features of habitat is increasingly recognised as a new avenue for investigating cause-and-effect relationships in ecology [68] and for deepening our understanding of species spatial patterns [10]. Indeed, our results demonstrate that our measure of fine-scale habitat heterogeneity may be useful for categorising suitability of habitat for the bush rat and long-nosed bandicoot, and other potentially heterogeneity-sensitive species (e.g. Long-nosed Potoroo [*Potorous tridactylus*]; [15]).

### How does fine-scale habitat heterogeneity influence occupancy?

As expected and based on the habitat heterogeneity hypothesis [4, 5], we found a positive effect of fine-scale habitat heterogeneity on species occupancy. We found that the long-nosed bandicoot and the bush rat were more likely to occur at sites with high levels of fine-scale small tree heterogeneity, and that the long-nosed bandicoot may be less likely to occur at sites with high levels of cover of small trees (4–10 m high; Fig 2). Our findings suggest that just as some species are adapted to dense habitats or to more open habitats, other species have adapted to take advantage of heterogeneity in the cover of habitat attributes [10].

Although a relationship between fine-scale habitat heterogeneity and species occupancy has not been previously explicitly tested for and established, past studies suggested such a relationship existed. For example, our findings for the long-nosed bandicoot concur with the observation of Bennett [69] that higher numbers of this species appear to occur in locally heterogeneous areas (i.e. sites with both dense understorey and open areas). In contrast, no previous studies have suggested that the bush rat is adapted to heterogeneous habitat. However, a study by Spencer et al. [70] found that the density of the bush rat was greatest at intermediate levels of mid-story cover. At intermediate levels of vegetation cover, the potential for high levels of heterogeneity of vegetation cover is highest [49]. Thus bush rat density in the study by Spencer et al. [70] also may be influenced by heterogeneity in mid-story cover or a correlated environmental attribute that these heterogeneous places offer, rather than cover *per se*.

At first glance, the influence of heterogeneity of cover of small trees seems unlikely to provide any direct benefit to ground-dwelling, terrestrial vertebrates, such as bush rats and long-nosed bandicoots. It therefore seems likely that this vegetation attribute was correlated with some other environmental factor that is of greater direct relevance to ground-dwelling animals. Occupying these highly heterogeneous areas would be advantageous to these species, if areas which are highly heterogeneous in terms of small tree cover provide higher abundance and greater variety of foraging resources (e.g. fungi, plants, fruit and invertebrates) than homogenous areas. Alternatively, the relationship with small tree heterogeneity and occupancy could reflect an adaptive trade-off between foraging needs and the need to avoid aerial predatiors (e.g. raptors) [14, 69, 71].

### How does ecotone proximity influence occupancy?

As we hypothesised on the outset of this investigation, we found that the probability of black wallaby occupancy was higher near to an ecotone (Fig 2). We suggest that the black wallaby is likely to be utilising the contrasting habitats surrounding an ecotone to meet their daily resource requirements and/or exhibiting behavioural trade-offs between predator avoidance (e.g. foxes [*Vulpes vulpes*]) and foraging behaviour. In the context of our study, an ecotone provides both dense habitat for shelter and predator avoidance, as well as feeding habitat [72, 73]. Other small macropod species have been shown to trade-off increased foraging benefits to remain close to protective vegetation cover [74]. Foraging resources for the black wallaby (plants and fungi; [73]) also may be higher closer to an ecotone. For instance, a higher density and diversity of plants (i.e. shrubs and herbs) and fungi can occur at ecotones, because different vegetation communities can co-occur in this transition zone [31, 75, 76]. A preference for ecotones has been reported for a number of other small to medium-sized macropod species, including the red-necked pademelon (*Thylogale thetis*), rufous hare-wallaby (*Lagorchestes hirsutus*), the red-necked wallaby (*Macropus rufogriseus*), and the bridled nail-tail wallaby (*Onychogalea fraenata*) [47]. These findings, and our own results, suggest that ecotones are an important habitat resource for small to medium-sized macropods.

Surprisingly, we found no effect of the distance from an ecotone for either the bush rat or long-nosed bandicoot. This suggests that these species have a preference for particular types of heterogeneous habitat (i.e. small tree heterogeneity) rather than heterogeneous edge habitat *per se*. Many studies have difficulty teasing apart habitat and edge effects [77]. However, our study emphasises how these two different factors can have different effects on different species.

### How does broad-scale habitat heterogeneity influence occupancy?

Despite a body of literature demonstrating the role of landscape heterogeneity (defined as the number of habitat types in our study) as a key determinant of species diversity responses [78–80], and in contrast to our hypothesis, we found no evidence of a relationship (negative or positive) between landscape heterogeneity and species occupancy, regardless of the spatial scales measured (i.e. within a 50, 100 and 150m radius of each site; Table 2). Our findings indicate that our study species were resilient to variation in landscape heterogeneity within our naturally heterogeneous landscape. For our study species, the majority of native habitats are neither inhabitable nor a barrier to movement, and an increase in the number of habitat types does not necessarily result in an increase in resources. Our findings highlight how similar to different aspects of fire mosaics [81], not all landscape heterogeneity elements may have functional roles for the various animal species [82].

### Implications of our results for management

While the maintenance of homogenous habitat is important for conserving some biota and communities [7, 10], we found that some small mammals, such as the long-nosed bandicoot and the bush rat, preferred fine-scale heterogeneous habitat. Our findings highlight the importance of fine-scale habitat heterogeneity to facilitate the persistence of these and other heterogeneity sensitive species. Our findings of a positive relationship between mammal occupancy and vegetation heterogeneity may be extended to threatened species and therefore indicate the vegetation heterogeneity should be considered when assessing the habitat requirements of threatened species. Promoting vegetation heterogeneity would be important in areas which have demonstrated a loss of fine-scale heterogeneity over time (e.g. associated with changing fire regimes) and an associated decline in fauna or for a threatened species that has identified heterogeneity as an important feature and where managers want to increase its area of occupancy. Furthermore, we found that different mammal species responded to different forms of vegetation heterogeneity. These findings suggest that management strategies that focus solely on managing only a single form of vegetation heterogeneity may disadvantage particular mammal species. For example, vegetation thinning to reduce vegetation cover homogeneity, may benefit heterogeneity-sensitive mammal species by increasing tree canopy heterogeneity at the fine-scale [83], but may have no beneficial effects for species that respond to other forms of heterogeneity, such as the black wallaby that was associated with ecotones. Other management actions, such as prescribed burning, can influence many forms of heterogeneity. For example, prescribed burning can result in both an increase in edge habitat [84], and an increase in vegetation heterogeneity in areas with topographically variable terrain [85, 86]. An understanding of the different ecosystem processes driving the different forms of vegetation heterogeneity can be used to determine which management strategies to use to benefit or disadvantage different species.

## Supporting Information

S1 Table The four broad categories of vegetation (forest, woodland, shrubland, and heathland) and 29 vegetation sub-formation classes used in our study. We identified four broad categories of vegetation (forest, woodland, shrubland, and heathland) and 29 vegetation sub-formation made up of different vegetation communities in our study. Full profiles of each of the 29 vegetation sub-formation classes are provided in Taws (1997).
(DOC)

S2 Table The stratified design. Sampling was stratified by fire frequency (0–3years; 4–8 years), slope angle (low [0.24–3.56 degrees]) and vegetation type (forest, woodland and heathland). We randomly selected 96 sites (eight replicates per treatment).
(DOC)

S3 Table The explanatory variables, model-covariate, spatial scale of measurement, and a description of the measures used in the analysis. The explanatory variables in the generalised linear models consisted of detection and occupancy covariates.
(DOC)

S4 Table Data used in the analysis. Data underlying the response variables (occupancy of long-nosed bandicoots [LNB], Black wallaby [BW] and bush rat [BR]) and model-covariates used in the analysis.
(DOC)
