# Correction: Fine-Scale Habitat Heterogeneity Influences Occupancy in Terrestrial Mammals in a Temperate Region of Australia

**DOI:** 10.1371/journal.pone.0140802

**Published:** 2015-10-14

**Authors:** Ingrid Stirnemann, Alessio Mortelliti, Philip Gibbons, David B. Lindenmayer

Fig 2 is a duplicate of Fig 1. The authors have provided a corrected version of Fig 2 here.

**Fig 2:**
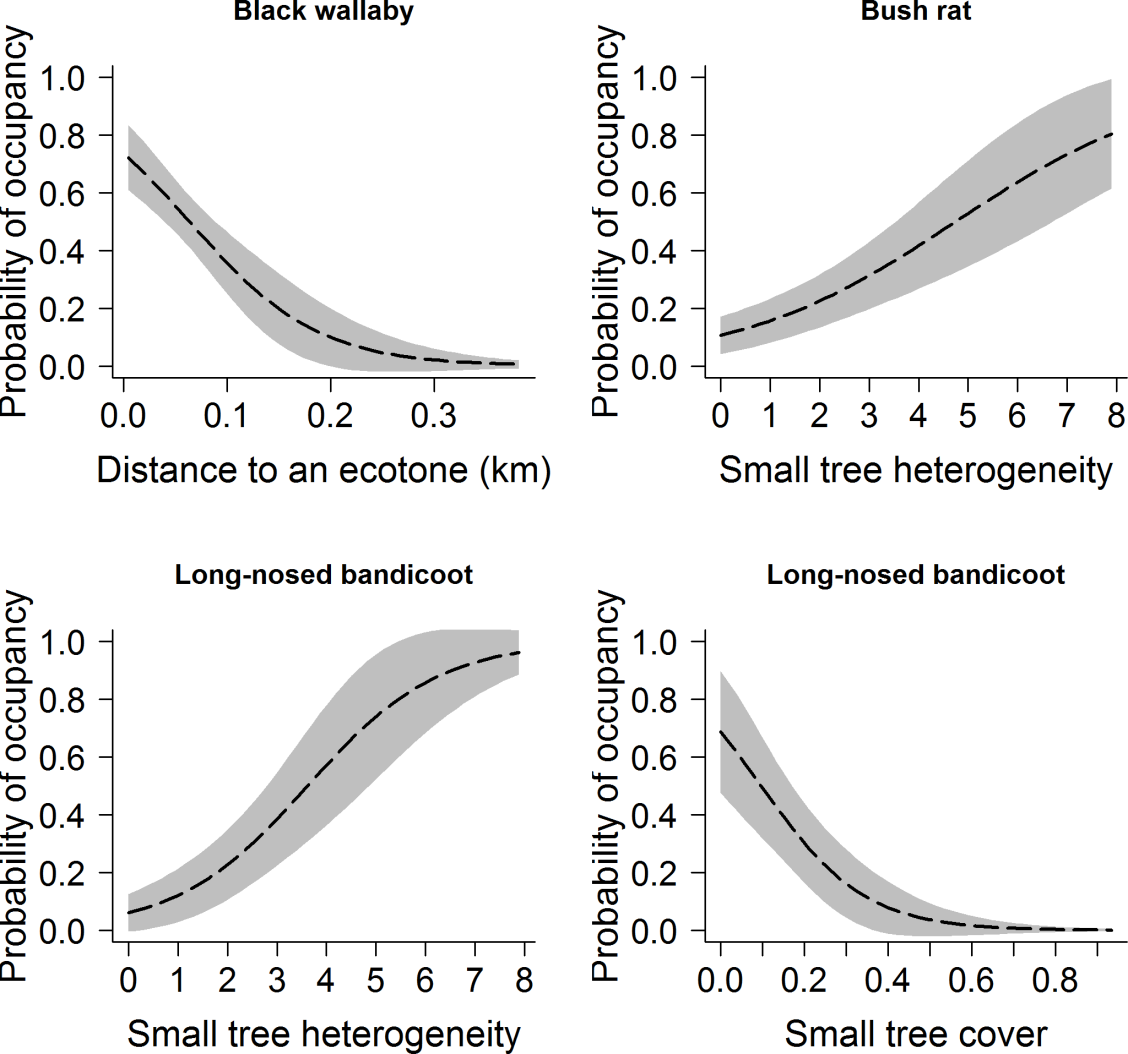
Probability of occupancy (± S.E.) of three mammals: black wallaby, bush rat, and long-nosed bandicoot in response to cover of small trees and heterogeneity of small trees, and distance to an ecotone.

## References

[1] Stirnemann I, Mortelliti A, Gibbons P, Lindenmayer DB (2015) Fine-Scale Habitat Heterogeneity Influences Occupancy in Terrestrial Mammals in a Temperate Region of Australia. PLoS ONE 10(9): e0138681 doi:10.1371/journal.pone.0138681 26394327 10.1371/journal.pone.0138681 PMC4579067

