# Supplementary material for: Fine-Scale Habitat Heterogeneity Influences Occupancy in Terrestrial Mammals in a Temperate Region of Australia
Source: PLoS One. 2015 Sep 22;10(9):e0138681. doi: 10.1371/journal.pone.0138681 (PMC4579067; doi:10.1371/journal.pone.0138681)
Supplement: S1 Table — We identified four broad categories of vegetation (forest, woodland, shrubland, and heathland) and 29 vegetation sub-formation made up of different vegetation communities in our study. Full profiles of each of the 29 vegetation sub-formation classes are provided in Taws (1997). (DOC) [file pone.0138681.s001.doc]

**S1 Table. The four broad categories of vegetation (forest, woodland, shrubland, and heathland) and 29 vegetation sub-formation classes used in our study**

**We identified four broad categories of vegetation (forest, woodland, shrubland, and heathland) and 29 vegetation sub-formation made up of different vegetation communities in our study. Full profiles of each of the 29 vegetation sub-formation classes are provided in Taws (1997).**

| Vegetation sub-formation | Broad vegetation categories |
| --- | --- |
| *Acacia sophorae*, *Leptospermum laevigatum* coastal shrubland | Shrublands |
| *Allocasuarina distyla* dry heath | Heathland |
| *Allocasuarina distyla* rocky heath | Heathland |
| *Allocasuarina distyla*, *Melaleuca capitata* rocky shrubland | Shrublands |
| *Allocasuarina* dry shrubland | Shrublands |
| *Allocasuarina verticillata* dry shrubland | Shrublands |
| *Avicennia marina* mangrove woodland | Woodlands |
| *Backhousia myrtifolia* dry rainforest | Forest |
| *Baeckea imbricata* coastal heath | Heathland |
| *Banksia ericifolia* intermediate heath | Heathland |
| *Banksia integrifolia* dry sclerophyll forest | Forest |
| *Banksia integrifolia* dry woodland | Woodlands |
| *Banksia serrata* dry woodland | Woodlands |
| *Casuarina glauca* swamp forest | Forest |
| *Ceratopetalum apetalum* warm temperate rainforest | Forest |
| Dry rainforest | Forest |
| *E.botryoides* swamp forest | Forest |
| *E.sclerophylla*, *E.gummifera* dry woodland | Woodlands |
| *Eucalyptus botryoides* dry sclerophyll forest | Forest |
| *Eucalyptus botryoides* swamp forest | Forest |
| *Eucalyptus botryoides* wet sclerophyll forest | Forest |
| *Eucalyptus gummifera* dry woodland | Woodlands |
| *Eucalyptus paniculata* dry sclerophyll forest | Forest |
| *Eucalyptus pilularis* dry schlerophyll forest | Forest |
| *Eucalyptus sclerophylla*, *E.gummifera* dry woodland | Woodlands |
| *Eucalyptus sieberi* , *E. gummifera* dry woodland | Woodlands |
| *Leptospermum laevigatum* coastal scrub | Shrublands |
| Littoral rainforest | Forest |
| *Sprengelia incarnata* intermediate heath | Heathland |
