# Supplementary material for: Fine-Scale Habitat Heterogeneity Influences Occupancy in Terrestrial Mammals in a Temperate Region of Australia
Source: PLoS One. 2015 Sep 22;10(9):e0138681. doi: 10.1371/journal.pone.0138681 (PMC4579067; doi:10.1371/journal.pone.0138681)
Supplement: S2 Table — Sampling was stratified by fire frequency (0–3years; 4–8 years), slope angle (low [0.24–3.56 degrees]) and vegetation type (forest, woodland and heathland). We randomly selected 96 sites (eight replicates per treatment). (DOC) [file pone.0138681.s002.doc]

**S2 Table. The stratified design**

Sampling was stratified by fire frequency (0-3years; 4-8 years), slope angle (low [0.24-3.56 degrees]) and vegetation type (forest, woodland and heathland). We randomly selected 96 sites (eight replicates per treatment).

|  | Fire frequency  (<3 years) | | Fire frequency  (4-8 years ) | |
| --- | --- | --- | --- | --- |
|  | Low slope | High slope | Low slope | High slope |
| Forest | 8 | 8 | 8 | 8 |
| Woodland | 8 | 8 | 8 | 8 |
| Heath | 8 | 8 | 8 | 8 |
|  |  |  |  | Total =  96 sites |
