# Supplementary material for: Fine-Scale Habitat Heterogeneity Influences Occupancy in Terrestrial Mammals in a Temperate Region of Australia
Source: PLoS One. 2015 Sep 22;10(9):e0138681. doi: 10.1371/journal.pone.0138681 (PMC4579067; doi:10.1371/journal.pone.0138681)
Supplement: S3 Table — The explanatory variables in the generalised linear models consisted of detection and occupancy covariates. (DOC) [file pone.0138681.s003.doc]

**S3 Table. The explanatory variables, model-covariate, spatial scale of measurement, and a description of the measures used in the analysis. The explanatory variables in the generalised linear models consisted of detection and occupancy covariates.**

| Explanatory variable | Model co-variate | | Scale | Description |
| --- | --- | --- | --- | --- |
| Heterogeneity of shrub cover | Occupancy | Fine-scale habitat variables | | Continuous |
| Heterogeneity of small trees | Occupancy | Fine-scale habitat variables | | Continuous |
| Heterogeneity of tall trees | Occupancy | Fine-scale habitat variables | | Continuous |
| Shrub cover | Occupancy | Fine-scale habitat variables | | Continuous |
| Small tree cover | Occupancy | Fine-scale habitat variables | | Continuous |
| Tall tree cover | Occupancy | Fine-scale habitat variables | | Continuous |
| Number of habitat types within a 50m radius of the sample points | Occupancy | Broad-scale habitat variables | | Continuous |
| Number of habitat types within a 100m radius of the sample points | Occupancy | Broad-scale habitat variables | | Continuous |
| Number of habitat types within a 150m radius of the sample points | Occupancy | Broad-scale habitat variables | | Continuous |
| Distance to ecotone (m) | Occupancy | Broad-scale habitat variables | | Continuous |
| Vegetation type (e.g. tall tree cover) | Detection | Fine-scale habitat variables | | Continuous |
| Total shrub cover | Detection | Fine-scale habitat variables | | Continuous |
| Heterogeneity of shrub cover | Detection | Fine-scale habitat variables | | Continuous |
| Camera type (Reconyx or Scoutguard) | Detection |  | | Categorical |
