# Supplementary material for: Fine-Scale Habitat Heterogeneity Influences Occupancy in Terrestrial Mammals in a Temperate Region of Australia
Source: PLoS One. 2015 Sep 22;10(9):e0138681. doi: 10.1371/journal.pone.0138681 (PMC4579067; doi:10.1371/journal.pone.0138681)
Supplement: S4 Table — Data underlying the response variables (occupancy of long-nosed bandicoots [LNB], Black wallaby [BW] and bush rat [BR]) and model-covariates used in the analysis. (DOC) [file pone.0138681.s004.doc]

**S4 Table. Data used in the analysis**

Data underlying the response variables (occupancy of long-nosed bandicoots [LNB], Black wallaby [BW] and bush rat [BR]) and model-covariates used in the analysis.

| **Heterogeneity** | | | **Cover** | | |  | **Number of habitat types** | | | **Occupancy** | | |
| --- | --- | --- | --- | --- | --- | --- | --- | --- | --- | --- | --- | --- |
| **shrub** | **mtree** | **Ltree** | **Ltree** | **mtree** | **shrub** | **Distance to an ecotone** | **50** | **100** | **150** | **LNB** | **BW** | **BR** |
| 3.97 | 3.40 | 5.61 | 0.41 | 0.24 | 0.63 | 61.6 | -1 | -1 | 3 | 0 | 1 | 0 |
| 1.26 | 1.59 | 5.88 | 0.40 | 0.07 | 0.56 | 12.3 | 0 | -1 | 4 | 0 | 0 | 0 |
| 3.58 | 2.01 | 0.00 | 0.00 | 0.88 | 0.25 | 44.1 | -1 | -1 | 4 | 0 | 1 | 0 |
| 1.91 | 0.80 | 3.54 | 0.32 | 0.03 | 0.77 | 18.9 | -1 | -1 | 5 | 0 | 0 | 0 |
| 1.70 | 5.37 | 2.36 | 0.05 | 0.24 | 0.76 | 16.8 | 0 | -1 | 4 | 1 | 1 | 0 |
| 1.04 | 0.54 | 5.73 | 0.37 | 0.01 | 0.89 | 24.2 | -1 | 0 | 3 | 0 | 0 | 0 |
| 2.22 | 4.87 | 3.54 | 0.19 | 0.32 | 0.52 | 65.3 | -1 | -1 | 3 | 1 | 0 | 0 |
| 2.21 | 2.07 | 2.47 | 0.40 | 0.25 | 0.60 | 52.7 | -2 | 0 | 3 | 1 | 1 | 0 |
| 2.21 | 1.18 | 1.27 | 0.49 | 0.57 | 0.32 | 25.9 | 0 | 0 | 3 | 0 | 0 | 0 |
| 2.30 | 2.42 | 4.84 | 0.41 | 0.12 | 0.75 | 30.0 | 0 | 0 | 3 | 0 | 1 | 0 |
| 1.59 | 5.47 | 0.89 | 0.04 | 0.33 | 0.83 | 18.2 | 0 | 0 | 3 | 0 | 1 | 0 |
| 3.32 | 2.08 | 2.90 | 0.08 | 0.41 | 0.28 | 82.5 | -1 | -1 | 3 | 0 | 0 | 0 |
| 1.46 | 2.78 | 0.00 | 0.00 | 0.28 | 0.79 | 15.4 | -1 | 0 | 3 | 0 | 0 | 1 |
| 2.61 | 1.27 | 0.00 | 0.00 | 0.04 | 0.79 | 35.2 | 0 | -1 | 3 | 0 | 1 | 1 |
| 2.94 | 0.00 | 5.49 | 0.75 | 0.00 | 0.55 | 25.0 | 0 | 0 | 3 | 0 | 0 | 1 |
| 0.55 | 2.57 | 2.26 | 0.55 | 0.11 | 0.40 | 16.8 | -1 | 0 | 4 | 0 | 0 | 0 |
| 3.12 | 4.62 | 5.00 | 0.33 | 0.09 | 0.64 | 72.2 | -1 | -1 | 3 | 1 | 0 | 1 |
| 4.25 | 1.93 | 6.07 | 0.55 | 0.15 | 0.51 | 28.4 | 0 | -2 | 4 | 0 | 1 | 0 |
| 1.61 | 3.55 | 0.00 | 0.00 | 0.77 | 0.49 | 49.2 | -1 | -1 | 4 | 0 | 0 | 1 |
| 2.09 | 1.98 | 6.09 | 0.23 | 0.32 | 0.68 | 54.8 | -2 | -1 | 4 | 0 | 0 | 0 |
| 2.66 | 4.25 | 3.72 | 0.11 | 0.21 | 0.51 | 14.0 | 0 | 0 | 3 | 0 | 0 | 0 |
| 3.82 | 3.17 | 3.62 | 0.20 | 0.29 | 0.31 | 39.1 | -2 | 0 | 4 | 0 | 1 | 0 |
| 3.06 | 2.17 | 4.45 | 0.56 | 0.08 | 0.28 | 72.9 | -1 | -1 | 3 | 1 | 0 | 0 |
| 2.81 | 3.95 | 5.29 | 0.13 | 0.60 | 0.24 | 24.3 | -2 | 0 | 4 | 0 | 1 | 0 |
| 0.72 | 1.11 | 0.00 | 0.00 | 0.88 | 0.35 | 21.9 | -1 | -1 | 4 | 0 | 1 | 0 |
| 2.91 | 7.65 | 0.57 | 0.01 | 0.41 | 0.48 | 16.5 | 0 | -1 | 5 | 1 | 1 | 1 |
| 0.83 | 1.77 | 1.92 | 0.56 | 0.16 | 0.05 | 37.7 | -1 | 0 | 4 | 0 | 0 | 0 |
| 2.67 | 1.50 | 3.60 | 0.41 | 0.29 | 0.57 | 22.3 | 0 | 0 | 2 | 0 | 1 | 0 |
| 2.03 | 1.82 | 0.00 | 0.00 | 0.60 | 0.59 | 26.5 | 0 | -1 | 3 | 0 | 1 | 0 |
| 3.08 | 3.26 | 0.00 | 0.00 | 0.15 | 0.79 | 84.2 | -1 | -1 | 3 | 0 | 0 | 1 |
| 0.54 | 2.36 | 0.00 | 0.00 | 0.05 | 0.69 | 28.7 | 0 | 0 | 2 | 0 | 1 | 0 |
| 1.39 | 0.00 | 0.00 | 0.00 | 0.00 | 0.83 | 24.2 | -2 | 0 | 4 | 0 | 0 | 0 |
| 1.17 | 0.00 | 0.00 | 0.00 | 0.00 | 0.85 | 198.6 | 0 | 0 | 1 | 0 | 0 | 0 |
| 1.03 | 0.57 | 0.00 | 0.00 | 0.01 | 0.92 | 48.3 | 0 | -1 | 3 | 0 | 0 | 0 |
| 2.20 | 1.72 | 1.72 | 0.05 | 0.04 | 0.88 | 22.0 | 0 | 0 | 2 | 0 | 1 | 0 |
| 4.96 | 0.00 | 0.00 | 0.00 | 0.00 | 0.43 | 36.8 | 0 | 0 | 2 | 0 | 0 | 0 |
| 2.13 | 4.12 | 3.61 | 0.11 | 0.11 | 0.72 | 9.0 | 0 | 0 | 2 | 1 | 1 | 0 |
| 10.06 | 0.00 | 1.52 | 0.07 | 0.00 | 0.60 | 68.7 | -2 | 0 | 3 | 0 | 0 | 0 |
| 0.83 | 1.27 | 0.00 | 0.00 | 0.04 | 0.95 | 104.5 | 0 | -2 | 3 | 0 | 0 | 0 |
| 0.83 | 0.00 | 0.00 | 0.00 | 0.00 | 0.95 | 103.4 | 0 | -2 | 3 | 0 | 0 | 0 |
| 0.99 | 2.52 | 0.80 | 0.03 | 0.05 | 0.92 | 70.7 | -1 | 0 | 2 | 1 | 1 | 0 |
| 1.59 | 0.00 | 0.00 | 0.00 | 0.00 | 0.93 | 63.1 | -1 | 0 | 2 | 1 | 1 | 0 |
| 1.35 | 0.00 | 0.00 | 0.00 | 0.00 | 0.89 | 51.5 | -1 | -1 | 3 | 0 | 0 | 0 |
| 1.59 | 0.00 | 0.00 | 0.00 | 0.00 | 0.89 | 41.8 | 0 | 0 | 2 | 0 | 0 | 0 |
| 0.81 | 0.00 | 0.00 | 0.00 | 0.00 | 0.87 | 45.4 | 0 | -1 | 3 | 0 | 1 | 0 |
| 3.73 | 1.40 | 0.00 | 0.00 | 0.08 | 0.52 | 38.2 | 0 | -1 | 3 | 0 | 1 | 0 |
| 1.59 | 0.00 | 0.00 | 0.00 | 0.00 | 0.93 | 32.2 | 0 | 0 | 2 | 0 | 1 | 0 |
| 4.28 | 4.64 | 0.00 | 0.00 | 0.37 | 0.51 | 128.9 | 0 | -1 | 2 | 0 | 0 | 0 |
| 1.95 | 3.94 | 0.00 | 0.00 | 0.24 | 0.81 | 26.4 | 0 | -2 | 4 | 0 | 0 | 0 |
| 2.40 | 2.52 | 0.00 | 0.00 | 0.56 | 0.72 | 45.0 | 0 | 0 | 3 | 0 | 0 | 1 |
| 5.14 | 1.37 | 0.00 | 0.00 | 0.08 | 0.77 | 19.8 | 0 | 0 | 2 | 0 | 0 | 0 |
| 1.54 | 0.00 | 0.00 | 0.00 | 0.00 | 0.83 | 22.2 | 0 | -1 | 3 | 1 | 0 | 0 |
| 2.42 | 7.58 | 0.00 | 0.00 | 0.32 | 0.88 | 14.7 | 0 | 0 | 3 | 1 | 0 | 1 |
| 6.29 | 3.29 | 0.00 | 0.00 | 0.15 | 0.79 | 63.8 | -1 | 0 | 2 | 0 | 0 | 0 |
| 3.28 | 0.00 | 0.00 | 0.00 | 0.00 | 0.84 | 39.7 | 0 | -1 | 3 | 0 | 0 | 1 |
| 0.90 | 0.00 | 0.00 | 0.00 | 0.00 | 0.88 | 39.4 | 0 | 0 | 2 | 0 | 0 | 0 |
| 1.00 | 0.00 | 0.00 | 0.00 | 0.00 | 0.65 | 80.3 | -1 | 0 | 2 | 1 | 1 | 0 |
| 0.32 | 1.72 | 0.00 | 0.00 | 0.05 | 0.59 | 15.9 | -1 | 0 | 4 | 0 | 0 | 0 |
| 1.96 | 1.20 | 0.00 | 0.00 | 0.04 | 0.80 | 65.5 | -1 | -1 | 3 | 0 | 0 | 0 |
| 2.28 | 0.00 | 0.00 | 0.00 | 0.00 | 0.85 | 82.9 | 0 | -2 | 3 | 0 | 1 | 0 |
| 2.08 | 5.28 | 4.43 | 0.72 | 0.65 | 0.24 | 27.3 | 0 | 0 | 2 | 0 | 1 | 0 |
| 4.44 | 1.56 | 3.52 | 0.75 | 0.12 | 0.35 | 210.2 | 0 | 0 | 1 | 0 | 0 | 0 |
| 2.71 | 4.89 | 1.60 | 0.84 | 0.36 | 0.29 | 79.1 | -1 | -1 | 3 | 0 | 0 | 0 |
| 1.21 | 1.60 | 0.00 | 0.00 | 0.79 | 0.05 | 82.4 | -1 | 0 | 2 | 0 | 0 | 0 |
| 0.86 | 1.56 | 7.36 | 0.56 | 0.07 | 0.04 | 89.0 | -1 | -1 | 3 | 0 | 0 | 0 |
| 2.89 | 1.11 | 1.21 | 0.48 | 0.03 | 0.17 | 136.8 | 0 | -1 | 2 | 0 | 0 | 0 |
| 7.31 | 2.12 | 4.81 | 0.48 | 0.12 | 0.43 | 70.5 | -1 | -1 | 3 | 0 | 0 | 1 |
| 2.96 | 0.74 | 2.25 | 0.67 | 0.03 | 0.51 | 81.5 | -1 | 0 | 2 | 0 | 0 | 0 |
| 1.00 | 2.28 | 2.61 | 0.68 | 0.28 | 0.47 | 34.6 | 0 | 0 | 2 | 0 | 0 | 0 |
| 4.27 | 4.42 | 6.06 | 0.31 | 0.16 | 0.48 | 139.9 | 0 | -1 | 2 | 0 | 0 | 0 |
| 0.77 | 2.30 | 4.89 | 0.79 | 0.08 | 0.03 | 158.6 | 0 | 0 | 1 | 1 | 0 | 0 |
| 1.11 | 0.00 | 0.00 | 0.78 | 0.05 | 0.03 | 55.0 | -1 | -1 | 3 | 0 | 1 | 0 |
| 1.86 | 3.92 | 1.79 | 0.91 | 0.15 | 0.12 | 145.8 | 0 | -1 | 2 | 0 | 1 | 0 |
| 1.49 | 1.18 | 0.00 | 0.00 | 0.03 | 0.79 | 64.0 | -1 | 0 | 2 | 0 | 1 | 0 |
| 0.57 | 2.94 | 6.28 | 0.65 | 0.11 | 0.01 | 137.2 | 0 | -1 | 2 | 1 | 0 | 0 |
| 1.27 | 7.89 | 6.60 | 0.52 | 0.36 | 0.04 | 62.5 | -1 | 0 | 2 | 0 | 0 | 0 |
| 2.19 | 7.16 | 10.32 | 0.52 | 0.31 | 0.13 | 41.8 | -1 | 0 | 3 | 0 | 0 | 1 |
| 2.38 | 4.63 | 1.47 | 0.84 | 0.16 | 0.16 | 33.3 | 0 | -1 | 3 | 0 | 0 | 0 |
| 0.86 | 5.10 | 5.74 | 0.57 | 0.28 | 0.04 | 39.0 | 0 | 0 | 2 | 0 | 1 | 0 |
| 1.59 | 1.41 | 2.31 | 0.91 | 0.15 | 0.44 | 50.7 | -1 | 0 | 2 | 0 | 0 | 0 |
| 4.11 | 3.05 | 5.91 | 0.68 | 0.12 | 0.51 | 44. | -2 | 0 | 4 | 0 | 0 | 0 |
| 0.57 | 5.33 | 5.53 | 0.75 | 0.20 | 0.01 | 92.5 | -1 | 0 | 2 | 0 | 0 | 0 |
| 2.25 | 4.88 | 2.14 | 0.93 | 0.28 | 0.67 | 180.3 | 0 | 0 | 1 | 0 | 1 | 0 |
| 1.83 | 5.25 | 7.03 | 0.41 | 0.48 | 0.04 | 200.1 | 0 | 0 | 1 | 0 | 0 | 1 |
| 2.35 | 2.08 | 3.05 | 0.93 | 0.93 | 0.33 | 286.8 | 0 | 0 | 1 | 0 | 0 | 0 |
| 2.22 | 4.87 | 6.61 | 0.69 | 0.23 | 0.23 | 56.3 | -1 | -1 | 3 | 0 | 1 | 0 |
| 2.22 | 3.12 | 2.59 | 0.79 | 0.27 | 0.53 | 114.7 | 0 | -3 | 4 | 1 | 0 | 0 |
| 3.87 | 1.73 | 4.63 | 0.72 | 0.16 | 0.17 | 44.0 | 0 | -1 | 3 | 1 | 0 | 0 |
| 1.24 | 2.73 | 4.54 | 0.65 | 0.67 | 0.04 | 87.1 | -1 | 0 | 2 | 0 | 0 | 0 |
| 2.33 | 4.89 | 0.00 | 0.00 | 0.60 | 0.71 | 4.7 | 0 | 0 | 3 | 0 | 1 | 0 |
| 1.86 | 0.00 | 0.00 | 0.00 | 0.00 | 0.80 | 36.0 | 0 | -1 | 4 | 0 | 0 | 0 |
| 2.16 | 1.65 | 2.35 | 0.75 | 0.07 | 0.11 | 86.5 | 0 | 0 | 1 | 0 | 0 | 0 |
| 1.83 | 2.33 | 5.69 | 0.49 | 0.08 | 0.20 | 382.3 | 0 | 0 | 1 | 0 | 0 | 0 |
| 1.28 | 2.95 | 3.37 | 0.21 | 0.09 | 0.17 | 54.3 | -1 | -1 | 3 | 1 | 0 | 0 |
